# The Role of Chronic Inflammation in the Development of Breast Cancer

**DOI:** 10.3390/cancers13153918

**Published:** 2021-08-03

**Authors:** David N. Danforth

**Affiliations:** Surgery Branch, Center for Cancer Research, National Cancer Institute, National Institutes of Health, Bethesda, MD 20892, USA; david_danforth@nih.gov; Tel.: +1-240-760-6213

**Keywords:** chronic inflammation, normal breast tissue, breast cancer, breast cancer development, obesity, microbiome, sterile inflammation

## Abstract

**Simple Summary:**

Chronic inflammation is an important cause of multiple cancers. While chronic inflammation is present in breast cancer and may influence its outcome, its role in the initiation and development of breast cancer is unclear. A review of the literature was conducted to determine if chronic inflammatory processes are present, both systemically and in normal breast tissue, which may contribute to the development of breast cancer in women. This indicates that several chronic inflammatory factors may influence breast cancer development, with some such as adipose tissue and obesity occurring early in breast carcinogenesis, while others, such as the microbiome and inflammation from genomic changes, may occur with the transition to malignancy. Chronic inflammation appears to be an important risk factor for breast cancer and may influence both the development and conduct of breast cancer.

**Abstract:**

Chronic inflammation contributes to the malignant transformation of several malignancies and is an important component of breast cancer. The role of chronic inflammation in the initiation and development of breast cancer from normal breast tissue, however, is unclear and needs to be clarified. A review of the literature was conducted to define the chronic inflammatory processes in normal breast tissue at risk for breast cancer and in breast cancer, including the role of lymphocyte and macrophage infiltrates, chronic active adipocytes and fibroblasts, and processes that may promote chronic inflammation including the microbiome and factors related to genomic abnormalities and cellular injury. The findings indicate that in healthy normal breast tissue there is systemic evidence to suggest inflammatory changes are present and associated with breast cancer risk, and adipocytes and crown-like structures in normal breast tissue may be associated with chronic inflammatory changes. The microbiome, genomic abnormalities, and cellular changes are present in healthy normal breast tissue, with the potential to elicit inflammatory changes, while infiltrating lymphocytes are uncommon in these tissues. Chronic inflammatory changes occur prominently in breast cancer tissues, with important contributions from tumor-infiltrating lymphocytes and tumor-associated macrophages, cancer-associated adipocytes and crown-like structures, and cancer-associated fibroblasts, while the microbiome and DNA damage may serve to promote inflammatory events. Together, these findings suggest that chronic inflammation may play a role in influencing the initiation, development and conduct of breast cancer, although several chronic inflammatory processes in breast tissue may occur later in breast carcinogenesis.

## 1. Introduction

Chronic inflammation has been recognized to play an important role in cancer since 1863 when Rudolph Virchow noted leucocytes in neoplastic tissues and suggested that the “lymphoreticular infiltrate” reflected the origin of cancer at sites of chronic inflammation [1]. Chronic inflammation is considered to be a contributing factor to the initiation and development of multiple cancers, including colorectal cancer, esophageal cancer, hepatocellular cancer, bladder cancer, gastric cancer, lung cancer, and others (reviewed and references in [2]). A range of etiologies are considered to be responsible for initiating and promoting the chronic inflammation for these cancers, including microorganisms (gastric cancer, colorectal cancer, bladder and hepatocellular cancer), gastric acid, tobacco, alcohol (esophageal cancer), tobacco products (lung cancer), asbestos (mesothelioma), and UV light (melanoma). Breast cancer is the most common malignancy and the second leading cause of cancer death in women in the United States [3]. While the importance of chronic inflammation in the development of other malignancies has been demonstrated, the role of chronic inflammation in the initiation and development of breast cancer is less clear.

There is evidence to indicate that chronic inflammatory changes are associated with breast cancer risk. The C-reactive protein (CRP), an acute phase protein, is considered to be a classic marker for inflammation [4]. CRP has been identified in nipple aspirate fluid (NAF) of healthy women and has been positively related to breast cancer risk as predicted by the Gail model [5]. The administration of anti-inflammatory agents, such as aspirin, to healthy women has been associated with a reduced risk for breast cancer [6]. In addition, crown-like structures, (CLS) a hallmark of chronic inflammation, have been identified in adipose tissue of the breast in obese women and are associated with an increased risk for breast cancer [7]. Normal breast tissue from healthy women also contains multiple immune cells, including CD4+ and CD8+ T lymphocytes, macrophages and dendritic cells, which could potentially mediate chronic inflammatory changes [8]. Together, these findings suggest chronic inflammatory changes may be associated with normal breast tissue and influence the risk for breast cancer.

Chronic inflammation is present in breast cancer and may be associated with multiple cellular changes including tumor infiltrating lymphocytes, tumor associated macrophages, crown-like structures of adipocytes, cancer-associated fibroblasts, and the tumor cells themselves. These chronic inflammatory actions may be triggered by products from microorganisms such as pathogen-associated molecular patterns (PAMPS) in the breast cancer, or cellular products released from injured or necrotic cells, such as damage-associated molecular patterns (DAMPS) and mediated by cytokines and chemokines secreted from associated innate or adaptive immune cells. These chronic inflammatory changes in breast cancer may have important consequences, both locally with tumor promotion and immunosuppression, as well as antitumor and cytotoxicity effects, and on outcome, including survival. Chronic inflammation thus represents an important component of breast cancer and would appear to fulfill many of the criteria to be considered the seventh hallmark of cancer as it is for other malignancies [9]. These observations suggest chronic inflammation may be present throughout breast carcinogenesis and influence multiple points in the initiation, development and behavior of breast cancer. Identification of the presence of these events at different stages of breast carcinogenesis would thus have important implications for both the prevention and treatment of breast cancer.

To address these issues, a review of the literature of chronic inflammatory processes in, or associated with, normal breast tissue and with breast cancer was conducted to clarify the course of chronic inflammation in breast carcinogenesis.

## 2. Materials and Methods

A literature search was conducted through PubMed, Google, and cross-references using the terms “normal breast tissue”, “breast cancer”, “chronic inflammation”, “inflammatory infiltrate”, “microbiome”, “adipocytes and obesity”, “crown-like structures”, “cancer-associated adipocytes”, “breast fibroblasts”, “breast cancer fibroblasts”, “mesenchymal stromal cells”, “sterile inflammation”, “proinflammatory”, and “anti-inflammatory” to identify publications describing the nature, distribution and mechanisms of inflammatory characteristics in normal breast tissue and in breast cancer. Normal breast tissue included tissue from biopsy, or reduction mammoplasty specimens, or a prophylactic mastectomy, as well as normal adjacent tissue or contralateral breast tissue in women with breast cancer. Breast cancer tissue included tissue from the breast cancer. Only articles written in English were included. Articles were published between 1999 and 2021 and included clinical and laboratory studies as well as selected reviews with expert interpretation and were from peer-reviewed journals. Articles were screened for analysis of the above parameters, relevance to chronic inflammation, risk to breast tissue and prognosis for breast cancer.

## 3. Chronic Inflammatory Changes Associated with Normal Breast Tissue

Normal breast tissue is a complex structure composed of multiple cell types, each with the potential to promote or inhibit chronic inflammation. These cell types include innate and adaptive immune cells (NK cells, CD4+ and CD8+ T cells, γδ T cells, macrophages, dendritic cells), adipocytes, fibroblasts, epithelial cells and the microbiome. Each of these cells may play a role in the inflammatory process. In addition, these cells are also in close proximity to regional draining lymph nodes (axillary, internal mammary) where dendritic cells from normal breast tissue may activate, for example, CD4+ cells to generate Th1, Th2, and Th17 cells, which can further influence inflammatory events. Normal breast tissue thus appears to provide the environment for chronic inflammatory changes to influence the development of breast cancer.

### 3.1. Evidence of Inflammation Associated with Breast Cancer Risk

There is evidence, both indirect and direct, that chronic inflammatory changes are associated with an increased risk for breast cancer in women. (A) Indirect studies include those demonstrating inflammatory changes which are reflected in plasma biomarkers such as C-reactive protein (CRP), and studies examining the effect of anti-inflammatory agents such as aspirin on risk for breast cancer. CRP is an acute phase protein considered to be a classic marker for inflammation [4]. A meta-analysis demonstrated that elevated plasma CRP levels were associated with an increased risk for breast cancer (OR, 1.22), with the association strongest in Asian women (OR, 1.57) [10]. Siemes et al. [11] observed high levels (>3 mg/L) of plasma CRP were associated with an increased risk of incident cancer (hazard ratio, 1.59–1.68). Obesity is an important risk factor for breast cancer [12,13,14], and plasma CRP levels are elevated in 7.7% and 20.2% of overweight and obese middle-aged women, respectively, compared with 4.8% in normal weight women [15]. Agnoli et al. [16] examined multiple inflammatory markers in plasma and found high CRP was significantly associated with increased breast cancer risk, and high adiponectin levels with a significantly reduced risk. Among premenopausal women, high TNF-α was associated with significantly increased risk, and high leptin with reduced risk. Additional systemic evidence of chronic inflammatory changes associated with breast cancer is provided by studies of the anti-inflammatory agent aspirin (ASA). When taken regularly, ASA has been shown to reduce the risk for breast cancer in women [17]. A recent meta-analysis suggested that aspirin may reduce the overall risk of breast cancer, reduce the risk of breast cancer in postmenopausal women, hormone-receptor-positive tumors, and in situ breast cancer [17]. Bertrand et al. [18] also reported a benefit of aspirin in ER negative and triple negative breast cancer in African American women. They concluded that the inverse associations of aspirin with ER negative and triple negative disease may therefore be driven by its anti-inflammatory effects rather than hormone-dependent mechanisms. (B) Direct studies include those measuring biomarkers in breast ductal fluid and in breast tissue and their relationship to breast cancer risk. Lithgow et al. [5] studied nipple aspirate fluid (NAF) for the presence of CRP in healthy women aged 30–65 years and found CRP was present and positively related to breast cancer risk as predicted by the Gail model. In that study, the serum CRP was not elevated, and thus the presence of CRP in NAF did not simply reflect its presence systemically but was felt to come from the breast epithelium. The relation between the intraductal levels of CRP in NAF and the 5-year Gail risk scores was considered to reflect a general schema that risk is reflected by an internal state of inflammatory changes in the mammary microenvironment. Hanna et al. [19] examined breast tissue for the presence of inflammatory markers in women with mammographically dense breast tissue, a known risk factor for breast cancer [20]. They found higher expression levels of the proinflammatory markers CRP, IL-6, TNF-α, and IL-8 were associated with a higher percent mammographic density (PMD) among premenopausal women, and higher expression levels of interleukin 6 were associated with higher PMD among all women (24.1% vs. 18.5%, *p* = 0.007). Higher expression levels (above median) of the anti-inflammatory marker TGF-β were associated with lower PMD among all women and among postmenopausal women. They concluded that these findings constitute further evidence that inflammation plays a role in breast cancer. The proinflammatory gene COX-2, a key driver of chronic inflammation [21], is induced by a variety of inflammatory stimuli, and its expression results in synthesis of prostaglandins with subsequent induction of the inflammatory response. Studies have demonstrated that mammographic breast density is due, in part, to increased deposition of collagen, and COX-2 expression correlates with mammographic density [22]. Collagen deposition in the microenvironment of breast cancer is also significantly associated with high stromal expression of COX-2 and CD163 macrophages [23]. The COX-2 gene and immunoreactive proteins have also been shown to be highly expressed and elevated in adipose tissue (AT) under morbid obesity conditions, another important risk factor for breast cancer [24]. Lastly, a recent study examined the breast ducts of women at normal risk and at high risk for breast cancer by ductal endoscopy. In this study the presence of intraductal fibrous webbing and stranding, an indicator of chronic inflammation, was observed in 40.4% of the ducts of women at high risk but only 5.4% of ducts of normal risk women [25]. Together, these findings provide evidence for the association of chronic inflammation in women and the risk for breast cancer.

### 3.2. Immune Cells and Inflammatory Cell Infiltrates in Normal Breast Tissue

**Figure 1:**
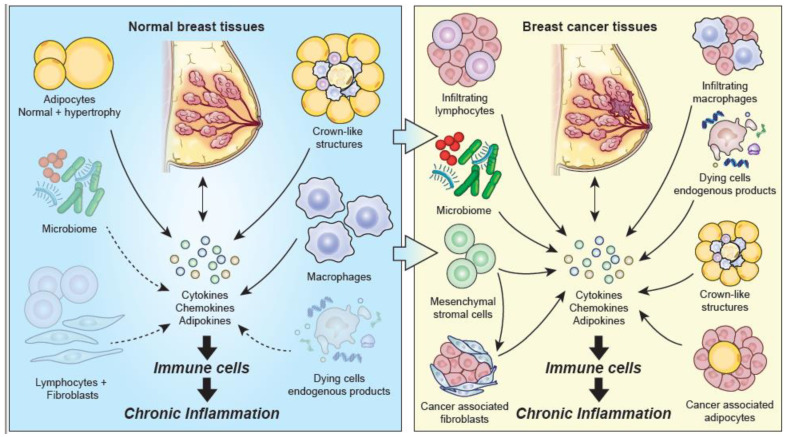
Chronic inflammatory processes in normal breast tissue and breast cancer tissue. Normal Breast tissue (left panel): Chronic inflammation may be initiated in the normal breast from several cell types. Hypertrophied adipocytes and crown-like structures are the most prominent focus and secrete multiple proinflammatory cytokines, including chemokines, and adipokines which promote chronic inflammation. The microbiome, dying or injured cells, and lymphocytes and fibroblasts, are shaded to indicate they are present in normal breast tissue, but their role in promoting chronic inflammation may occur later in breast carcinogenesis. Breast cancer tissue (right panel): Multiple cellular processes are present in breast cancer promoting chronic inflammation. Infiltrating lymphocytes and macrophages, cancer-associated adipocytes and crown-like structures, mesenchymal stem cells and cancer-associated fibroblasts that secrete multiple proinflammatory cytokines, including chemokines and adipokines, while the microbiome and dying or injured cells interact with immune cells through MAMPS and DAMPS to promote chronic inflammation (DN Danforth, original figure).

Normal breast tissue contains multiple cell types including innate (dendritic cells, macrophages, NK cells) and adaptive (CD4+, CD8+, B cells) immune cells, adipocytes and fibroblasts (Figure 1) [8].

Immune cells are present in breast tissue but do not appear to be infiltrative on the order of inflammatory lymphocyte or macrophage infiltrates such as are seen in breast cancer. Degnim et al. [26] described CD8+, CD11c+, CD45+, and CD68+ cells, with lower densities of CD4+ and CD20+, which were predominantly localized to the lobules rather than the stroma of normal breast tissue. Ruffell et al. [27] found myeloid-lineage cells including macrophages (CD14^hi^CD11b+HLADR+), mast cells (FcεR1α+CD117+CD11b−CD49d+) and neutrophils (CD15+CD11b+CD49d−) were more evident in normal tissues (both cancer-adjacent and prophylactic mastectomy), which contrasted with that in breast cancer where the density of immune infiltrates was substantially increased and consisted of infiltrates dominated by T lymphocytes (CD3ε+), with minor populations of natural killer cells (CD3ε−CD56+NKG2D+) and B lymphocytes (CD19/20+HLA-DR+CD3−). Hussein et al. [28] observed lower quantities of CD3+, CD20+, CD68+ and granzyme+ cells in normal breast stroma and parenchyma but substantially increased quantities in breast cancer. They considered the presence of mononuclear inflammatory cell infiltrate in the normal breast tissues to reflect ongoing immune responses against the neoplastic cells. These studies of normal breast tissue, however, did not describe an increase in macrophages, Th1 or Th17 inflammatory CD4+ cells, or other inflammatory markers that might suggest the presence of chronic inflammatory changes. Two other studies have been conducted of benign normal breast conditions, one regarding lobulitis and a second examining benign breast disease. Degnim et al. [26] studied patients with lobulitis and noted significantly higher cell densities of CD4+, CD8+, CD20+, and CD45 cell types in the lobules with lobulitis compared to lobules without lobulitis, while the densities of innate immune cells (monocytes/macrophages and dendritic cells) did not vary significantly with the presence of lobulitis. The authors noted that the immune cell infiltrates of lobulitis were present in the normal condition and that immune infiltrates were not necessarily pathologic but may represent a higher immune cell density present in almost all normal lobules. Degnim et al. [29] also described an immune cell density in patients with benign breast disease (BBD, all of which had evidence of histologic abnormality including proliferative changes with or without atypia). They found BBD lobules showed greater densities of CD8+ T cells, CD11c+ dendritic cells, CD20+ B cells, and CD68+ macrophages compared with normal controls. The increased immune cell infiltration observed in BBD tissues relative to normal mammary gland tissue suggested there was a local immune response which may be antigen-specific. The finding that macrophages were more common in lobules of BBD than in normal tissues also suggested greater inflammation in tissues with higher cancer risk. Together, these findings would suggest that tissues with BBD histologic abnormalities, especially proliferative disease, may be accompanied by increased immune cell infiltrates, but that inflammatory infiltrates are otherwise uncommon in normal breast tissue.

### 3.3. Inflammatory Changes of Adipose Tissue Associated with Maintaining Homeostasis and with Breast Cancer Risk

Adipose tissue is a complex structure composed of preadipocytes, adipocytes, macrophages, endothelial cells, fibroblasts and leukocytes [30,31]. White adipose tissue (WAT) is the predominant form in adults and, under normal conditions, serves to maintain homeostasis through secretion of multiple cytokines, adipokines and growth factors, which regulate a wide range of processes including immunity, angiogenesis, glucose and lipid metabolism, fibrinolysis, and body weight homeostasis [31]. These events may include proinflammatory changes involved in proper extracellular matrix remodeling and angiogenesis, two processes known to facilitate adipogenesis in vivo [32]. Adipose tissue inflammation under these conditions is considered an adaptive response that enables safe storage of excess nutrients and contributes to a visceral depot barrier that effectively filters gut derived endotoxins [32]. Adipose tissue in the healthy individual also contains several anti-inflammatory mechanisms and adipokines, including adiponectin, C1q/TNF-related proteins (CTRPs), omentin, and secreted frizzled-related protein 5 (SFRP5) [33]. Adiponectin, for example, abrogates LPS-stimulated TNF production by macrophages, inhibits Toll-like receptor (TLR)-mediated NF-κB activation in macrophages, and stimulates the production of the anti-inflammatory cytokine IL-10 by human macrophages [34]. Macrophages in lean adipose tissue are primarily M2 macrophages involved in downregulating inflammation and initiating wound repair through the release of anti-inflammatory cytokines such as IL-4, IL-10, IL-13, and TGFβ [33,35,36]. Anatomical studies have shown that the fat volume in the breast may occupy up to 56% of the total breast volume, providing an important source for local effects of adipose tissue [37].

Adipose tissue can also represent an important source of chronic inflammatory changes associated with increased risk for breast cancer, and these changes may occur in lean as well as overweight or obese tissue. A recent study examined the transcriptome in adipose tissue of the breast in normal healthy women [38]. They found expression of a previously defined active transcriptome phenotype which showed significantly larger adipocytes (*p* < 0.01) than those with the inactive phenotype. Expression of single genes associated with adipocyte activation (leptin, leptin receptor, adiponectin), proinflammatory fat signaling (IKBKG, CCL13), fat remodeling (CAV1, BNIP3), and adipokine growth factors (IGF-1, FGF2) were all significantly elevated in the active samples relative to the inactive samples. The subject population included lean as well as overweight or obese women, however subjects with the active phenotype had no difference in BMI but significantly higher Gail scores (1.46 vs. 1.18; *p* = 0.007). These findings indicated the presence of larger adipocytes, as well as a proinflammatory transcriptome in lean as well as overweight or obese women.

Adipose tissue, as it hypertrophies, secretes inflammatory cytokines and adipokines such as TNFα, IL-1, IL-6, IL-8, resistin and leptin, and attracts macrophages with the aid of chemokines including monocyte chemotactic protein (MCP-1), macrophage migration inhibition factor (MIF-1), macrophage inflammatory proteins (MIP-1α), chemokine CCL5 (RANTES) and others [31,34]. Obesity is associated with local inflammation and macrophage infiltration, with obese women having higher average macrophage counts and inflammatory foci counts than normal-weight women in normal human breast adipose tissues [39]. Resistin, a secretory product of adipocytes, is upregulated in obesity-induced inflammation and induces NF-κB activity and has proinflammatory and proliferative properties [40]. Leptin, an important secretory product of adipocytes that is increased in obesity, has important proinflammatory actions on the secretion of cytokines (TNFα, IL-1, IL-6) and on innate and adaptive immune cells [41]. Adipocytes express a functional and proinflammatory toll-like receptor (TLR) signaling system, which allows for the ability to respond rapidly to acute changes in whole body homeostasis, such as infection [42]. TLRs on adipocytes, including TLR 1, 2, 3, 4, 6, can respond to exogenous products such as lipopolysaccharide (LPS; from bacterial cell walls), stearic acid and palmitic acid to induce proinflammatory cytokine and chemokine release in adipocytes, stimulating the release of TNF-α, IL-1β, IL-6, IL-8, MCP-1, and resistin by activating the TLR–MD-2–NFκB pathway. Release of free fatty acids from adipocytes following lipolysis, with subsequent binding to TLR4 on macrophages, results in M1 activation of macrophages, which is associated with increased NF-κB binding activity and aromatase expression and activity [43,44]. This functional TLR pathway in adipocytes thus directly connects innate immunity with adipocyte function [42,45]. These actions and proinflammatory products are also in close proximity to ductal epithelial cells, and may promote chromosomal instability, increased proliferation, and potentially initiate breast carcinogenesis. Obesity is also characterized by enhanced levels of reactive oxygen or nitrogen species [46], which can cause genotoxic damage to epithelial cells and contribute to progression in the carcinogenic pathway [47]. At the same time obesity may also be characterized by a decrease in anti-inflammatory factors such as adiponectin, CTRP12 (Adipolin), omentin, and Sfrp5 [30,33]. Collectively, these anti-inflammatory factors normally act to block NF-kB activation and reduce cytokines TNFα, IL-6 and IL-18 [33], decrease macrophage accumulation and proinflammatory gene expression [48], inhibit TNFα-induced vascular inflammation [33,49], and control inflammatory cells within adipose tissue [50]. The proinflammatory changes have been well summarized by Van Kruijsdijk et al. [51] who observed that obesity is strongly associated with changes in the physiological function of adipose tissue, increased levels of leptin, plasminogen activator inhibitor-1, endogenous sex steroids, and chronic inflammation, which are involved in carcinogenesis and cancer progression.

Crown-like structures (CLS), a hallmark of chronic inflammation, may be present in normal breast tissue. A very nice study by Carter et al. [7], demonstrated that the incidence of CLS in breast tissue is related primarily to the presence or absence of benign breast disease (BBD) histologic changes (nonproliferative or proliferative changes). In the absence of BBD (or breast cancer), CLSs in the breast tissue of either lean or obese women are rare (incidence 0.0% and 6.3%, respectively; Table 1).

In the presence of BBD in normal breast tissue, CLSs are increased with increasing BMI (incidence = 13.3–24.2% at BMI < 30, and 45.8% in obese women), but are independent of age [7]. These data suggest that CLS-B (CLS of breast)–associated adipose tissue inflammation occurs in a significant subset of individuals with BBD, and that BBD stromal tissues are far more frequently inflamed than donor (lean) breast tissues [7]. These authors previously reported that lobules of BBD tissues had a significantly higher density of CD68-positive macrophages compared with lean individuals [29], supporting the hypothesis that BBD is associated with an epithelial and stromal chronic inflammatory environment. Importantly, high CLS-B densities were found to be independently associated with an increased breast cancer risk. Among BBD biopsies, a high CLS-B count (>5 CLS-B/sample) conferred a breast cancer OR of 6.8 (95% CI, 1.4–32.4), *p* = 0.02 [7].

Analyses of the stromal vascular and adipocyte fractions of the mammary gland have also suggested that macrophage-derived proinflammatory mediators induce aromatase and estrogen-dependent gene expression (PR, pS2) in adipocytes. The presence of CLS in breast tissue, and saturated fatty acids which have been linked to obesity-related inflammation, have been shown to stimulate NF-κB activity in macrophages leading to increased levels of TNF-α, IL-1β, and Cox-2, each of which contributed to the induction of aromatase in preadipocytes [57]. This process has been referred to as the obesity→inflammation→aromatase axis [57]. Aromatase functions to convert androgens to estrogens, and elevated levels of aromatase expression and activity could contribute to the increased incidence of hormone-receptor positive breast cancer in obese postmenopausal women, thus representing another important consequence of chronic inflammatory changes in breast tissue.

### 3.4. Fibroblasts in Normal Breast Tissue

In normal breast tissue fibroblasts are a major component of the extracellular matrix (ECM) and serve to produce collagens, reticular fibers and other components of the extracellular matrix. The stroma of the normal breast differs significantly from the stroma of breast cancer, containing sparse connective tissue surrounding the duct compared with abundant connective tissue in carcinoma [58]. There is a strong interaction between fibroblasts and epithelial cells which may be involved in carcinoma initiation and promotion, including secretion of the fibroblast growth factor (FGF) family, the IGF family, the EGF family, hepatocyte growth factor (HGF), and the TGF-β family [58]. Most of these factors are predominantly stimulators of proliferation and can play a role in promoting the carcinogenic process, whereas TGFβ is a growth inhibitor of breast epithelial cells [58]. The role of fibroblasts in chronic inflammation in normal breast tissue, however, is unclear. Parsonage et al. [59] described a Stromal Access Code consisting of multiple components including CXCL12, CXCL13, CCL19, CCL21, adhesion molecules, cytokines IL-6, IL-7, and fibroblast growth factors-7 (FGF-7, FGF-10), which have been shown to act to regulate leucocyte accumulation, differentiation, and survival in several stromal niches (bone marrow, thymus, lymph node). The aberrant expression of components of this code contributes to the persistence of chronic inflammatory disease at these sites [59]. It has also been reported that fibroblasts in hyperplastic or dysplastic skin lesions may contain a proinflammatory gene signature [60], including expression of chemokines and interleukins and which may be involved in early carcinogenesis [60,61]. These studies suggest a potentially important role for fibroblasts in chronic inflammation. Whether expression of this signature or the signal address code occurs in, for example, hyperplastic but otherwise normal breast tissue, is not known, but in view of the importance of fibroblasts to breast tissue structure and function, this would be an important question for future studies.

### 3.5. Potential Contributors to Chronic Inflammation in Normal Breast Tissue

In addition to the cellular components, there are other factors which may contribute to inflammation in normal breast tissue such as the microbiome and cellular substances released from injured or dying cells. Both of these factors are present in breast cancer tissues and play a role in promoting the chronic inflammatory changes in these tumors (see breast cancer discussion below). As a whole, the breast is a favorable environment for the growth of bacteria, as it is made up of fatty tissue with extensive vasculature and lymphatic drainage [62]. Healthy normal breast tissue contains a prominent microbiome [63,64,65]. The inflammatory changes associated with the microbiome in these tissues appear to serve primarily to maintain homeostasis. Commensal bacteria in healthy normal breast tissue are considered to play an important role in nutritive, immune-modulating and metabolic contributions for the maintenance of health [62,66]. Bacteria may maintain the healthy status of breast tissue by stimulating host inflammatory responses [62,67]. These bacteria and their metabolites can interact with TLRs of the innate immune system to trigger inflammatory signaling pathways to promote tissue repair and regeneration, and help maintain homeostasis [68], as well as limit or inhibit chronic inflammatory actions [69]. Macrophage activation may also result in secretion of a number of endogenous anti-inflammatory mediators including the cytokines IL-4, IL-10, IL-13, TGF-β, VEGF, and lipid mediators [35,70]. Chan et al. [71] has proposed that certain microbes might play a preventative role in breast carcinogenesis by affecting levels of estrogen or by promoting antitumor immunity and immune surveillance. Urbaniak et al. [72] observed that Lactococccus and Streptococcus were more prevalent in healthy women than in breast cancer patients. Both exhibit anticarcinogenic properties and may play a role in prevention. Lactococcus lactis can activate NK cells to produce IFN-γ which subsequently activates dendritic cells and macrophages, and production of the anti-inflammatory cytokine IL-10 [73]. De Moreno et al. [74] has also demonstrated that Lactobacillus helveticus stimulates production of the anti-inflammatory cytokines IL-4 and IL-10 (mediators of the anti-inflammatory macrophages M2) and decreases production of IL-6. At the same time, studies have compared normal cancer-adjacent breast tissue with healthy normal breast tissue and observed a prominent difference in the microbiome content [64,65,72]. The cancer-adjacent breast tissue microbiome is considered dysbiotic, with the potential to influence breast carcinogenesis. Interestingly, the genera *Fusobacterium* has been demonstrated in healthy normal breast tissue and was more abundant in the normal cancer-adjacent breast tissue [64,65,72]. The *Fusobacterium* genus may release factors and provide a proinflammatory environment, which leads to carcinogenesis [75]. This would indicate a potential source of chronic inflammatory events in healthy normal tissue. It is also important to note that studies have demonstrated that consumption of a Western or Mediterranean diet modulated mammary gland microbiota and metabolite profiles, and thus directly influenced microbiome populations in the mammary gland [76]. This would suggest that the composition of the microbiome in healthy normal breast tissue may be dynamic and vary according to dietary changes. At the same time, it is recognized that studies examining healthy normal breast tissue (or cancer-adjacent breast tissue) for the presence of chronic inflammatory changes associated with the microbiome have not been described, raising the possibility that these changes may occur later with the transition to malignancy.

The initiation of sterile inflammation by release of endogenous products from injured or dying cells is an important potential source of chronic inflammation, and there is evidence this process may occur in healthy normal breast tissue. Damaged or dead cells may release a range of endogenous substances (alarmins) including proteins and peptides, polysaccharides and proteoglycan, nucleic acids and phospholipids, which in turn may interact with TLRs on immune cells to regulate many sterile inflammatory processes [77]. A very interesting earlier study examined the morphological identification of cell multiplication (mitosis) and cell deletion (apoptosis) within the lobules of the “resting” human breast during the menstrual cycle [78]. They found that responses—mitosis and apoptosis—had a biorhythm in phase with the menstrual cycle, with a 3-day separation of the mitotic and apoptotic peaks. Both processes showed significant cyclical variation (*p* < 0.0001) with the peak of mitosis occurring at Day 25, and that for apoptosis occurring at Day 28. The 3-day separation of the peak in events was statistically significant. The study did not find significant differences in the responses between groups according to parity, contraceptive-pill use or presence of fibroadenoma. Of note, a recent study has shown that the high mobility group box 1 protein (HMGB1), an important endogenous protein [77], is released extracellularly during apoptotic cell death [79]. HMGB1 also has the potential to interact with several TLRs and induced sterile inflammation [77]. These findings provide a potential link between cellular activities and the initiation of inflammation in the resting breast, especially one which may occur early in breast carcinogenesis. In addition, normal breast cells contain a range of genomic changes [80], and damaged human cells can develop persistent chromatin lesions bearing hallmarks of DNA double-strand breaks (DSBs) which may initiate increased secretion of inflammatory cytokines such as interleukin-6 (IL-6) [81]. Sterile inflammation may thus be an important but less recognized source of inflammation in the healthy breast. Further studies are needed to define the presence and nature of inflammatory changes associated with these processes in normal breast tissue.

Lastly, there exists a variety of benign inflammatory conditions of the breast (mastitis), either infectious or noninfectious, which may, or may not, contribute to the chronic inflammation of breast carcinogenesis [82]. Some are more common (lactational mastitis, breast abscess, fibrocystic disease) than others (plasma cell mastitis, diabetic mastopathy). The significance of chronic inflammatory changes in these lesions is not clear. Some would be considered in the category of BBD described by Degnim et al. [83] (fibrocystic disease and ductal hyperplasia with/without atypia) and might have associated chronic inflammatory changes. Other inflammatory conditions, such as lactational mastitis or chronic granulomatous disease, are not generally considered to be associated with an increased risk for breast cancer.

## 4. Chronic Inflammatory Changes Associated with Breast Cancer

### 4.1. Chronic Inflammatory Cell Infiltrates in Breast Cancer Tissues

Breast cancer contains a prominent chronic inflammatory component consisting of cells of the immune system (lymphocytes, macrophages, dendritic cells, monocytes, neutrophils) as well as cancer-associated adipocytes, crown-like structures of adipocytes, and cancer-associated fibroblasts (Figure 1). Inflammatory cell infiltrates consisting of lymphocytes (CD4+ and CD8+ T cells and B+ cells) and macrophages are a common feature of breast cancer (Table 2; [27,84]).

Mohammed et al. [84], for example, observed that 91% of patients with invasive ductal carcinoma had high grade inflammatory immune cell infiltrates, with tumor lymphocytic infiltrate, macrophage infiltrate, CD8+ T-lymphocytic infiltrate, and B-lymphocytic infiltrate independently associated with cancer survival. Others [28] have found the inflammatory infiltrate to be primarily in the stroma, and to consist of CD3+ cells reflecting increased T cell proliferation. Ruffell et al. [27] also described a prominent immune cell infiltrate in breast cancer and dominated by T lymphocytes (CD3ε+), with minor populations of natural killer cells and B cells. They noted a shift within tumors toward a TH2-type response in BC characterized by increased presence of B cells and CD4+ T cells, in comparison with normal breast tissue. Activated dendritic cells may translocate bacterial antigens to regional lymph nodes to promote the activation of adaptive CD4+ T cells to Th1, and Th17 with associated inflammatory functions [108]. Secretion of IFN-γ and TNFα by Th1 cells may activate proinflammatory M1 macrophages [109]. Th17 cells may constitute up to 29.3% of breast cancer infiltrating lymphocytes in some patients [110]. Th17 produces cytokines IL-17, IL-21 and IL-22 [85]. The inflammatory cytokine IL-17A, for example, binds to breast cancer cells and activates oncogenic ERK and NF-κB pathways, and can bind to fibroblasts and activate NF-κB and STAT3 pathways leading to production of IL-6 and G-CSF. IL-6, in combination with TGFβ, further activates Th17 cells leading to a chronic inflammatory state and amplification of IL-17A signaling [111]. IL-17 and IL-21 may also serve to recruit polymorphonuclear lymphocytes. Interestingly, Eftekhari et al. [112] recently observed that Th1 and Th17 expression was reduced in breast cancer compared with healthy normal breast tissue, suggesting these cell types may also play a role in tumor rejection. CD8+ T cells in breast cancer are predominantly of the effector memory cell subtype, with cytotoxic capacity mediating antitumor immunity, but with the ability to secrete proinflammatory cytokines IL-2, TNFα, and INFγ as well [86,113]. Chronically activated B cells promote accumulation of innate cells in the neoplastic stroma by immunoglobulin and cytokine production [114]. Together, these proinflammatory mediators may have an important influence on all phases of breast carcinogenesis, including potentiation, tumor cell proliferation, transformation, invasion and metastasis [115].

Macrophages play an important role in chronic inflammation in breast cancer. Tumor-associated macrophages (TAMs) are among the most common cells in the leucocyte infiltrate and may constitute over 50% of the number of cells within the tumor [109]. Macrophages may be present as the classically activated M1 phenotype or the alternatively activated M2 phenotype; however, there is evidence that macrophages exhibit different phenotypes during different stages of tumor initiation and progression. During early stages of transformation, recently recruited macrophages are exposed to a wide variety of proinflammatory signals derived from the epithelial cells and the surrounding stroma, and often express M1-related factors that have protumorigenic properties, such as IL-1β and IL-6 [116]. M1 macrophages exhibit potent microbicidal and tumoricidal activity by releasing proinflammatory cytokines (such as TNF, IL-1, IL-6, IL-12, IL-23), promoting strong proinflammatory Th1 immune responses and exerting antiproliferative and cytotoxic activities, which result from the release of reactive oxygen species (ROS) and reactive nitrogen species (RNS) [35]. TNFα has been shown to be increased in breast cancer compared with healthy normal breast tissue [112]. In established, progressive breast cancers, IL-4 and IL-13 derived from Th2 cells elicit alternative M2 activation of TAMs [117], with production of immunosuppressive factors such as IL-10 and TGF-β that are capable of actively suppressing the antitumor immune response [116]. Most TAMs in the tumor microenvironment are closely related to the M2-like phenotype [118]. Chemokines, including CC chemokines, are major determinants of macrophage and lymphocyte infiltration in carcinoma of the breast [1].

Interestingly, several studies have demonstrated an increase in inflammatory infiltrates in the subtype hormone receptor-negative breast cancer, including high lymphocytic infiltrate, plasma cell infiltrate, other inflammatory cell infiltrate and macrophage infiltrate associated with ER neg/PR neg tumors [84], higher numbers of peri- and paratumoral tumor infiltrating B lymphocytes associated significantly with hormone receptor (ER/PR) negative (*p* = 0.008) and HER2+ status [119], and high granulin expressing bone marrow cells recruited to breast cancer expression and correlated with the most aggressive triple-negative, basal-like tumor subtype [120]. On the other hand, serum CRP, a marker of chronic inflammation, appears to be independent of tumor subtype, with elevations in receptor-positive as well as receptor-negative tumors [121,122,123,124].

### 4.2. Potential Contributors to Chronic Inflammation in Breast Cancer

Breast cancer contains a prominent microbiome which is considered dysbiotic and with the potential to influence breast carcinogenesis [67]. Microbiota may trigger inflammation through release of a variety of substances (MAMPS or PAMPS, such as dsRNA, LPS and lipopeptides) which react with pattern recognition receptors (PRRs) on innate immune cells [70,95]. Engagement of the PRRs on macrophages, for example, activates the macrophage to the M1 subtype, triggering signaling pathways that lead to the release of the inflammatory cytokines TNFα, IL-1, IL-12, IL-23 (Table 2 [35,108]), chemokines and the recruitment and activation of lymphocytes, with the propagation of chronic inflammation [35,70,108]. Bacterial antigen activation of CD4+ lymphocytes to Th1, Th2, and Th17 cells was described above. It has recently been shown, for example, that microbially driven TLR5-dependent IL-6 signaling promotes breast cancer malignant progression through tumor-promoting inflammation [125]. Li et al. [126] demonstrated that lipopolysaccharide (LPS), a major structural component of Gram-negative bacteria and a potent inducer of inflammation, promoted pulmonary metastases from breast cancer in an animal model.

Immune cells may also respond to endogenous ligands released from cells as a result of cell stress, injury or cell death, through a process called sterile inflammation [105,127,128]. Cell stress may result from genomic instability from mutational changes, inherited genomic changes and increased proliferation, as well as metabolic, hypoxic, or mechanical stress, including environmental carcinogens [128]. The DAMPS may be released by a variety of cells, although the majority of endogenous ligands appear to be extracellular matrix components generated as a result of tissue injury [77]. Many of the PRRs activate a shared set of inflammatory pathways, including NF-κB, p38, ERK, inflammasome assembly and IL-1β and IL-18 release, as well as secretion of IL-6, TNF, LT-β, IFNγ and TGF-β, and promote the recruitment of inflammatory cells [106]. A pathway for the activation of tumor antigen–specific T-cell immunity that involves secretion of the high-mobility-group box 1 (HMGB1) alarmin protein by dying tumor cells, and its action on Toll-like receptor 4 (TLR4) expressed by dendritic cells (DCs), has recently been described in breast cancer [129]. This pathway may also play an important role in the response to radiotherapy and chemotherapy. At the same time, while there are multiple proinflammatory events induced by factors released from dying cells, there is also evidence these events may be tempered to some degree by DAMPS released from these cells which are anti-inflammatory, reducing expression of cytokines IFN-β, IL-6 and IL-12 [130]. Multiple endogenous inhibitors of the pro-inflammatory transcription factor NF-κB have also been identified [131].

### 4.3. Cancer-Associated Adipocytes and Crown-Like Structures

Adipocytes constitute the main cellular component of the extracellular matrix in breast cancer [132]. These adipocytes, referred to as cancer-associated adipocytes (CAAs), are in close proximity to cancer cells and are morphologically and phenotypically distinct from adipocytes of normal breast tissue [132]. Adipocytes in breast cancer undergo reprograming, which involves the metabolic regulation of almost all macronutrients, such as carbohydrates, lipids, and amino acids [92]. CAAs secrete inflammatory factors that modify the behavior of breast cancer cells, including greater secretion (compared to normal adipocytes) of chemokines CCL2, and CCL5, interleukin-1β (IL-1β), interleukin-6 (IL-6), tumor necrosis factor-alpha (TNF-α), vascular endothelial growth factor (VEGF), leptin, adiponectin and IGF-1 [92]. The high CAA intratumoral adipocyte content has been shown to be enriched in inflammation-related gene sets such as TNF-α signaling via NF-κB, IL-6/JAK/STAT3 signaling and inflammatory response, and thus a high amount of intratumoral adipokines associated with inflammation that promote angiogenesis, invasion, metastases, and tumor progression [93]. At the same time, a high intratumoral adipocyte count may also be associated with less unfavorable immune cells including less type 2 helper T cells (Th2), regulatory T cells (Treg), and M2 macrophages (M2), especially in the ER+/Her2–subtype, suggesting a more favorable immune response [93]. This suggests that intratumoral adipocytes may contribute to breast cancer through inflammation, metastasis, and cancer stemness, but may also be associated with a favorable immune response that slows the cancer aggressiveness down, particularly in this subtype [93].

Macrophages recruited to adipose tissue bind to the adipocytes of dying cells at the CCR2 receptor to form crown-like structures (CLS) [7]. CLSs composed of macrophages (typically proinflammatory M1 type) are a histologic hallmark of the proinflammatory process and may be present in the adipose tissue near the breast cancer. Tumor-associated macrophages (TAMS) and CLS are more frequent in adipose tissue of breast cancer than normal (reduction mammoplasty) adipose tissue, and in breast cancer are more frequent in breast tissue of obese than of lean women (Table 1). Interestingly, whereas CLSs are rare in normal lean women, they are present in lean women with breast cancer (incidence 8.0–34.8%; Table 1). The finding of CLS in lean individuals indicates a subset of lean individuals that are known to be metabolically unhealthy [53]. Release of proinflammatory mediators, including TNF-α, IL-1, IL-6, and cyclooxygenase-2 (COX-2)-derived prostaglandin E2 (PGE2), contribute to the chronic inflammatory state and may also act to upregulate the transcription of the *CYP19* gene encoding aromatase, leading to estrogen production [57]. Proinflammatory macrophages might also promote malignant transformation through release of mutagenic reactive oxygen and reactive nitrogen species. TAMs and CLS contribute to the increased risk and worse prognosis of breast cancer. High levels of CD163+ macrophages were associated with shorter disease-free survival in node-negative breast cancer patients (*p* = 0.033), and CD68+ CLSs were associated with shorter overall survival in node-positive breast cancer patients (*p* = 0.015) [133]. It has also recently been shown that breast cancers from black patients contain significantly higher densities of TAMs and CLS compared with non-black Latinas and Caucasian women [134]. However, whether and how immune inflammatory components in the tumor microenvironment (TME) may correlate with the aggressiveness of breast cancer in AA women remains unclear and is an important topic for future studies [134].

### 4.4. Mesenchymal Stromal Cells (MSCs) and Cancer-Associated Fibroblasts

Mesenchymal stromal cells (MSCs) are adult, fibroblast-like multipotent cells characterized by the ability to differentiate into tissues of mesodermal origin, such as adipocytes, chondroblasts, osteoblasts [99], and, importantly, into cancer-associated fibroblasts [135]. MSC may arise from a variety of sites, including adipose tissue, skin, tendon, muscle, dental pulp and others [99,136]. MSC niches respond to numerous signals generated by the tumor milieu, including TGF-β, IL-6, Cyclophilin B, HDGF, uPA/uPAR, MCP-1, VEGF and FGF2, which can act as chemoattractants for MSCs [137]. At the tumor site, MSCs infiltrate into the stroma and produce bioactive molecules such as CCL5, IL-6, SDF-1 and TGFβ, which promote tumor growth and/or distant metastasis to secondary organs, such as the lungs [137]. Within the tumor, the behavior of MSC may be determined by the inflammatory nature of the environment. As nicely summarized by Bernado et al. [99], in the absence of an inflammatory environment (low levels of TNFα and IFNγ), MSCs may adopt a proinflammatory phenotype (MSC1) and enhance T cell responses by secreting chemokines that recruit lymphocytes to sites of inflammation (e.g., MIP-1a and MIP-1b, RANTES, CXCL9, CXCL10, and CXCL11) [100,138]. Polarization to a proinflammatory MSC1 phenotype can also be influenced by activation of TLR4 by low levels of lipopolysaccharide (LPS) derived from Gram-negative bacteria, suggesting an important interaction with microbia in breast tissue [99]. In the presence of an inflammatory environment (high levels of TNFα and IFNγ), MSCs become activated and adopt an immune-suppressive phenotype (MSC2) by secreting high levels of soluble factors, including IDO, PGE2, NO, TGFβ, Hepatocyte Growth Factor (HGF) and hemoxygenase (HO), that suppress T cell proliferation [99,101]. Importantly, potent proinflammatory cytokines (tumor necrosis factor and interleukin 1) lead to the conversion of mesenchymal stem cells (MSCs) to inflammatory cancer-associated fibroblasts (CAFs) [135]. Rubenstein, et al. [135] have shown that these inflammation-driven CAFs secrete metastasis-promoting factors that elevate the dispersion, scattering, and migration of breast cancer cells via activation of tumor cell receptors that signal through Ras proteins and via GαI proteins; the latter receptors were identified as the chemokine receptors CCR2, CCR5, and CXCR1/2. MSCs are thus highly versatile cells with the ability to respond to a spectrum of stimuli and with the potential to influence multiple aspects of breast carcinogenesis.

Cancer-associated fibroblasts (CAFs) are also an important contributor to the inflammatory component of breast cancers. CAFs are the largest population of stromal cells in breast tumors [139] and are considered to represent up to 80% of stromal fibroblasts. CAFs are phenotypically and metabolically distinct from normal fibroblasts [140,141,142]. Genetic and/or epigenetic cancer-specific alterations have been observed in CAFs, including defective p53/p21-dependent signaling pathway, low basal levels of p53 and p21, and strong expression of the proliferation markers Ki-67 [143,144]. CAFs are implicated in every stage of cancer from initiation to colonization, and one of the most important roles of CAFs is to mediate cancer inflammation, which is a major driver of metastasis [143]. A proinflammatory gene signature has been identified in CAFs of breast tumors and consists of two chemokines that are chemoattractants for neutrophils and macrophages (CXCL1, and CXCL2), the proinflammatory cytokines IL-1β and IL-6, the proangiogenic gene CYR61, COX-2, and osteopontin (OPN), which is known to affect inflammation, angiogenesis and metastasis [60,143,145]. By the secretion of chemokines and cytokines, CAFs attract inflammatory cells including macrophages, monocytes, and neutrophils to the tumor [143]. Activation of CAFs in triple negative breast cancer, for example is associated with expression of CXCL16, which is a monocyte attractant [145]. CAFs can activate the NFκB signaling pathway to evoke a proinflammatory response, and through the secretion of IL-1β, IL-6, IL-8, and SDF-1 induce the recruitment of immune cells and influence epithelial behavior [102]. Cytokines from CAFs and from mononuclear inflammatory cells have also been shown to have prognostic significance: a high score of IL-6, and the expression of IL-1β by each stromal cells (CAFs and MICs) was significantly associated with both longer relapse free-survival (RFS) and overall survival (OS) [146]. CAFs thus play an important role in the chronic inflammatory events in breast cancer, influencing the activities of both epithelial cells, immune cells, extracellular matrix and other components of the tumor microenvironment.

### 4.5. Chronic Inflammation and the Promotion of Metastatic Disease

Breast cancer is clearly a systemic disease, and an important consequence of chronic inflammation in breast cancer is the potential for promotion of metastatic disease. This has been emphasized in several publications discussed above and may be associated with proinflammatory activities of multiple cell types including adipocytes [93], MSC [137], and CAFs [143], or from associated immune cells. A very interesting recent study demonstrated a possible mechanism for the promotion of micrometastases by inflammation. It was shown that the systemic inflammatory and immunosuppressive response to surgery triggers the outgrowth of distant immune-controlled tumors in mouse models of dormancy [147]. Panigrahy et al. [148] addressed this concept in a mouse model and demonstrated that the preoperative administration of the anti-inflammatory agent ketorolac (an NSAID) eradicated micrometastases and promoted long-term survival in multiple tumor-resection models. The antitumor effect of ketorolac was T cell-dependent and involved increases in CD4+ and CD8+ cells and a reduction of FOXP3+ cells, which was augmented by immune checkpoint blockade, negated by adjuvant chemotherapy and dependent on inhibition of the COX-1/thromboxane A2 (TXA2) pathway. Thromboxane A2 (TXA2) downstream from COX-1 is a potent vasoconstrictor and induces platelet aggregation, a process that is known to help metastatic tumor seeding and cancer cell survival in the mouse. TXA2 production was decreased with preoperative ketorolac. This study thus provides compelling evidence for the manner in which chronic inflammation in tumors may promote the development of metastatic disease.

## 5. Summary and Conclusions

Chronic inflammation may play an important role throughout breast carcinogenesis, influencing the initiation, development and outcome of breast cancer. In early breast carcinogenesis, obesity (a prevalent problem with an incidence of 43% of women across all age groups [149]) is an important contributor through adipocyte hypertrophy and crown-like structures, especially in the presence of histologic evidence of benign breast disease. Other factors, such as the microbiome, genomic abnormalities and cellular changes, are present in healthy breast tissue with the potential to elicit inflammatory changes. Together, however, the inflammatory processes and changes in normal breast tissue appear to be less than in those of breast cancer, suggesting many of these changes may occur later, or with the transition to malignancy. The importance of chronic inflammation in the development of breast cancer encourages further studies and the identification of new related targets for breast cancer prevention.

## Figures and Tables

**Table 1:** Incidence of crown-like structures in breast adipose tissue according to body weight status.

Normal Breast Tissue							
Lean, (BMI < 25)	Overweight (BMI 25–29.9)	Obese	BBD BMI < 30 Controls ^2^	BBD BMI < 30 Cases ^3^	BBD Obese Controls ^2^	BBD Obese Cases ^3^	References
0.0% ^1^	3.3% ^1^	6.3% ^1^	6.3–8.0%	13.3–24.2%	45.8%	45.0%	[7]
**Breast Cancer Tissues**							
17%		54%					[52]
34%	53%	90%					[53]
8%	70%	75%					[43]
	57.0% ^4^						[54]
24%	34%	57% ^5^ 65% ^6^					[55]
34.8%	50%	100%					[56]

^1^ Komen Tissue Bank; ^2^ BBD—breast tissue with benign breast disease, Control—no future breast cancer; ^3^ BBD, Cases—future breast cancer; ^4^ Median BMI 25.7. ^5^ Obesity, 30–35 kg/m^2^; ^6^ Obesity ≥ 35.0 kg/m^2^.

**Table 2:** Cellular proinflammatory and anti-inflammatory substances.

Cell Type	Proinflammatory Cytokines, Chemokines, Growth Factors	Anti-Inflammatory Cytokines, Chemokines, Growth Factors	References
Infiltrating lymphocytes	Cytokines IL-2, TNFα, and INFγ as well, IL-17, IL-21 and IL-22.		[85,86]
Adipocyte	IL-1β, IL-6, TNF-α, resistin, leptin, MCP-1, CCL2, CXCL8, CXCL1, CXCL10	Adiponectin, CTRPs, SFRP5, omentin, TGFβ	[31,33,34,87]
Adipocyte in obesity	TNF, IL-1β, IL-6, IL-12, IL-17, IL-18, IFN-γ, resistin and leptin, RBP4, lipocalin 2, CCL2, CCL5, CXCL5, MCP-1, MIP-1α)	Adiponectin, CTRPs, Omentin, SFRP5, IL-10	[31,33,88,89,90]
Crown-like structures	TNF, IL-1β, IL-6, and leptin, CCL2		[33,91]
Cancer associated adipocyte	Chemokines CCL2 and CCL5, IL-1β, IL-6, TNF-α, VEGF, leptin	Adiponectin	[92,93,94]
Microbiome	MAMPS, PAMPS, microbial metabolites	Short-chain fatty acids, IL-10, TGFβ	[70,95,96]
M1 macrophages	TNFα, IL-1, IL-6, IL-12, IL-23, CXCL9, CXCL10, ROS, RNS		[35,97,98]
M2 macrophages		IL-4, IL-10, IL-13, TGFβ, VEGF, EGF	[35,98]
Mesenchymal stem cells	MIP-1a and MIP-1b, RANTES, CXCL9, CXCL10, CXCL11	IL-6, HGF, TGFβ, IL-1RA, PGE2, Galectin-1, IDO, NO, PDL-1	[99,100,101]
Fibroblasts	FGF, IGF, EGF, HGF, TGF-β family		[58]
Cancer-associated fibroblasts	IL-1, IL-6, IL-8, IL-10, TNFα, CXCL1, CXCL2, CXCL5, CXCL12, CCL2, CCL5, COX2		[60,102,103,104]
Dying/Injured cells	Endogenous agents including proteins and peptides, polysaccharides and proteoglycans, nucleic acids, phospholipids, or small organic molecules.		[77,105,106,107]

RBP-4—retinol binding protein; CTRPs-C1q/TNF—related proteins; SFRP5—secreted frizzled-related protein5; MCP-1—monocyte chemoattractant protein-1; IDO—indoleamine 2,3-dioxygenase; IL-1RA-IL-1 receptor antagonist; NO—nitric oxide; MIP-1α—macrophage inflammatory proteins; FGF—fibroblast growth factor; IGF—insulin-like growth factor; EGF—epidermal growth factor; HGF—hepatocyte growth factor; PGE2—prostaglandin E_2_; MAMPS—microbe associated-molecular patterns; PAMPS—pathogen-associated-molecular patterns; ROS—reactive oxygen species; RNS—reactive nitrogen species.

## Data Availability

Not applicable.

## Abbreviations

ASA—aspirin; CRP—C-reactive protein; DAMPS—damage-associated-molecular patterns; HMGB1—high-mobility-group box 1; MAMPS—microbe-associated-molecular patterns; NSAID—nonsteroidal anti-inflammatory drug; NAF—nipple aspirate fluid; PAMPS—pathogen-associated-molecular patterns; PRR—pattern recognition receptors; TLR—toll-like receptors; CLS—crown-like structures; RR—relative risk; CAA—cancer associated adipocytes; CAF—cancer associated fibroblasts; TNB—triple negative breast cancer; MSC—mesenchymal stem cells.
